# Supplementary material for: Sodium and Potassium Intake, Knowledge Attitudes and Behaviour Towards Salt Consumption Amongst Adults in Podgorica, Montenegro
Source: Nutrients. 2019 Jan 13;11(1):160. doi: 10.3390/nu11010160 (PMC6356471; doi:10.3390/nu11010160)
Supplement: Supplementary file 1 [file nutrients-11-00160-s001.pdf]

Table S1. Supplemental Table 1.

Variable	All (n = 639)	Excluded * (n = 351)	p-value
Age (yrs)	46.8 (11.3)	44.8 (11.2)	0.008
Height (cm)	175.4 (9.2)	175.8 (9.4)	0.5
Weight (kg)	79.8 (16.8)	80.4 (17.3)	0.6
B.M.I. (kg/m^2)	25.8 (4.2)	25.8 (4.6)	1.0
Systolic BP (mmHg)	125.1 (16.2)	127.0 (15.4)	0.08
Diastolic BP (mmHg)	79.1 (9.2)	80.2 (9.0)	0.07
Pulse rate (b/min)	72.4 (8.7)	73.8 (10.0)	0.02

* Participants without complete urines (see Figure 2)

Table S2. Daily urinary excretions of volume, sodium, potassium and creatinine by age (n = 639).

	I (n = 118)	II (n = 239)	III (n = 282)
Volume (mL/24 hour)	1465 (535)	1560 (576)	1755 (638) *
Sodium (mmol/24 hour)	181.0 (78.9)	189.5 (96.8)	186.2 (89.3)
Potassium (mmol/24 hour)	59.6 (23.2)	63.1 (26.0)	63.1 (27.4)
Creatinine (g/24 hour)	1.48 (0.60)	1.47 (0.57)	1.33 (0.51) *

Results are mean (SD); * p for trend: $p < 0.01$.

Table S3. Estimates of salt and potassium intake by age (n = 369).

	I (n = 118)	II (n = 239)	III (n = 282)
Salt intake (g/day)	11.3 (4.9)	11.8 (6.0)	11.6 (5.6)
Potassium intake (g/day)	3.0 (1.2)	3.2 (1.3)	3.2 (1.4)

Results are mean (SD).

Table S4. Knowledge, attitudes and behaviours towards salt consumption by age.

Question	I (n = 107)	II (n = 224)	III (n = 268)
Do you add salt to food at the table? (Often/Always) (%)	76.1	73.5	73.1
In the food you eat at home salt is added in cookin (Often/Always) (%)	24.8	23.9	17.9
How much salt do you think you consume? (Too much) (%)	36.8	39.1	32.1
Do you think that a high salt diet could cause a serious health problem? (Yes) (%)	83.8	85.7	86.8
How important is lowering salt/sodium in your diet? (Very important) (%)	41.4	41.6	47.5
Do you do anything on a regular basis to control your salt/sodium intake? (Yes) (%)	30.2	33.5	36.8

Supplemental Table S5. Daily urinary excretions of sodium and potassium by education (*n* = 622).

	Low (up to Secondary school) *n* = 158	Medium (High school) *n* = 237	High (University degree) *n* = 227
Sodium (mmol/24 hour)	190.9 (93.3)	199.4 (90.0)	171.0 (85.8) *
Potassium (mmol/24 hour)	59.0 (25.0)	63.9 (26.7)	63.9 (26.6)

**p* = 0.002 vs Medium

Supplemental Table S6. Daily urinary excretions of sodium and potassium by work (*n* = 599).

	Government employee *n* = 195	Non-government employee *n* = 188	Other * *n* = 95	Retired *n* = 80	Unemployed *n* = 41
Sodium (mmol/24 hour)	178.3 (87.3)	192.7 (87.5)	165.6 (91.5)	193.3 (75.4)	216.1 (191.0) **
Potassium (mmol/24 hour)	61.4 (26.3)	65.8 (26.9)	57.8 (27.8)	63.2 (25.9)	62.2 (25.4)

* Self-employees, Non-paid, Student, Homemaker; ***p* = 0.02 vs Other
